# Impact of ventilator settings during venovenous extracorporeal membrane oxygenation on clinical outcomes in influenza-associated acute respiratory distress syndrome: a multicenter retrospective cohort study

**DOI:** 10.7717/peerj.14140

**Published:** 2022-10-10

**Authors:** Ting-Yu Liao, Sheng-Yuan Ruan, Chien-Heng Lai, Li-Jung Tseng, Li-Ta Keng, You-Yi Chen, Chih-Hsien Wang, Jung-Yien Chien, Huey-Dong Wu, Yih-Sharng Chen, Chong-Jen Yu, TACTICS Study Group

**Affiliations:** 1 Departments of Internal Medicine, National Taiwan University Hospital Hsin-Chu Branch, Hsin-Chu, Taiwan; 2 Departments of Internal Medicine, National Taiwan University Hospital, Taipei, Taiwan; 3 Department of Surgery, National Taiwan University Hospital, Taipei, Taiwan; 4 Department of Internal Medicine, National Taiwan University Hospital Yun-Lin Branch, Dou-Liu, Taiwan; 5 Thoracic Medicine Center, Department of Medicine and Surgery, National Taiwan University Hospital Yunlin Branch, Dou-Liu, Taiwan

**Keywords:** Extracorporeal membrane oxygenation, Influenza, Acute respiratory distress syndrome

## Abstract

**Background:**

Patients with influenza-associated acute respiratory distress syndrome (ARDS) requiring venovenous extracorporeal membrane oxygenation (vv-ECMO) support have a high mortality rate. Ventilator settings have been known to have a substantial impact on outcomes. However, the optimal settings of mechanical ventilation during vv-ECMO are still unknown.

**Methods:**

This multicenter retrospective cohort study was conducted in the intensive care units (ICUs) of three tertiary referral hospitals in Taiwan between July 2009 and December 2019. It aims to describe the effect of ventilator settings during vv-ECMO on patient outcomes.

**Results:**

A total of 93 patients with influenza receiving ECMO were screened. Patients were excluded if they: were receiving venoarterial ECMO, died within three days of vv-ECMO initiation, or were transferred to the tertiary referral hospital >24 hours after vv-ECMO initiation. A total of 62 patients were included in the study, and 24 (39%) died within six months. During the first three days of ECMO, there were no differences in tidal volume (5.1 *vs.* 5.2 mL/kg, *p* = 0.833), dynamic driving pressure (15 *vs.* 14 cmH2O, *p* = 0.146), and mechanical power (11.3 *vs.* 11.8 J/min, *p* = 0.352) between survivors and non-survivors. However, respiratory rates were significantly higher in non-survivors compared with survivors (15 *vs*. 12 breaths/min, *p* = 0.013). After adjustment for important confounders, a higher mean respiratory rate of >12 breaths/min was still associated with higher mortality (adjusted hazard ratio = 3.31, 95% confidence interval = 1.10–9.97, *p* = 0.034).

**Conclusions:**

In patients with influenza-associated ARDS receiving vv-ECMO support, we found that a higher respiratory rate was associated with higher mortality. Respiratory rate might be a modifiable factor to improve outcomes in this patient population.

## Introduction

Influenza is a respiratory tract infection that is prevalent globally, affecting 1 billion adults every year (Global Influenza Strategy, 2019). Although most infected patients only experience mild illness, seasonal influenza epidemics result in approximately 3–5 million cases of severe illness annually  (Iuliano et al., 2018). In Taiwan, approximately 0.5% of influenza patients require hospitalization and 7% of patients hospitalized for influenza need intensive care (Influenza, 2014). Some hospitalized influenza patients may develop severe acute respiratory distress syndrome (ARDS) which requires venovenous extracorporeal membrane oxygenation (vv-ECMO) support.

Although vv-ECMO has been shown to be a promising treatment for patients with influenza-associated severe ARDS (Roch et al., 2010; Noah et al., 2011), the mortality of this disease is still high, ranging from 21% to 56% of patients (Roch et al., 2010; Davies et al., 2009; Pham et al., 2013). Vv-ECMO provides high-level gas exchange and oxygen support and enables clinicians to reduce ventilator settings to prevent further ventilator-induced lung injury from high mechanical forces (Schmidt et al., 2019; Abrams et al., 2020). However, a recent study of ECMO in ARDS showed that, despite most ECMO centers adopting ultra-protective lung ventilation, defined as a driving pressure ≤15 cm H2O and tidal volume of ≤4 ml/kg, this setting during ECMO did not significantly improve outcomes  (Schmidt et al., 2019). This unexpected finding may be related, in part, to a lack of defined optimal ventilator settings other than tidal volume during vv-ECMO (Schmidt et al., 2019; Abrams et al., 2020). Furthermore, a recent animal study (Araos et al., 2019) found that 24 h of near-apneic ventilation (respiratory rate of five breaths/min) significantly decreased histologic lung injury and fibroproliferation compared with both the nonprotective and protective lung strategies. Several previous experimental studies have also shown that decreasing respiratory rate may prevent ventilator-induced lung injury (Hotchkiss Jr et al., 2000; Retamal et al., 2016). Thus, we hypothesized that a lower mandatory respiratory rate during vv-ECMO in patients with ARDS might further reduce damage to the injured alveoli and result in better patient outcomes. In this multicenter study, we aimed to retrospectively investigate the association between mortality and mechanical ventilator settings, mechanical forces from mechanical ventilators, baseline characteristics and intensive care unit (ICU) management among patients with influenza-associated ARDS treated with mechanical ventilation and vv-ECMO support.

## Patients and Methods

### Population and study design

This study was conducted in the ICUs of three tertiary referral hospitals in Taiwan. All adult patients with influenza-related ARDS admitted to the participating ICUs between July 2009 and December 2019 were retrospectively identified. The diagnosis of influenza was confirmed by a lateral-flow immunoassay or quantitative real-time polymerase chain reaction (PCR). ARDS was diagnosed according to the Berlin definition (Force et al., 2012). Before 2012, ARDS was diagnosed using the prior diagnostic criteria defined by the 1994 consensus (Bernard et al., 1994). Patients who were intubated for mechanical ventilation accompanied by ECMO support were enrolled. The criteria for cannulation were: a ratio of arterial oxygen partial pressure to fractional inspired oxygen (PaO2/FiO2) <80 mmHg under optimal medication treatment or hypercapnic respiratory failure (pH <7.25) despite optimal mechanical ventilation (respiratory rate 35 breaths/min and plateau pressure ≤ 30 cmH2O). The criteria for preparing for decannulation was: an improvement in chest imagery with compliance >20 mL/cmH2O and tidal volume (TV) >6 ml/kg under peak inspiratory pressure (PIP) ≤ 30 cmH2O. Patients were excluded if they: (1) had clinically suspected myocarditis using venoarterial extracorporeal membrane oxygenation (va-ECMO), (2) died within 3 days of vv-ECMO initiation, or (3) were transferred to the tertiary referral hospital >24 h after vv-ECMO initiation. This study was performed in accordance with all relevant guidelines and regulations. The institutional ethics board of the National Taiwan University Hospital (202002073RINA) approved this study and waived the need for informed consent due to the retrospective nature of study.

### Data collection

We collected the following data from the medical records: demographics, comorbidities, Charlson comorbidity index scores (Charlson et al., 1987), laboratory data including arterial blood gas before vv-ECMO setup and 24 h after vv-ECMO setup, Sequential Organ Failure Assessment (SOFA) scores (Vincent et al., 1996) and Acute Physiology and Chronic Health Evaluation II (APACHE II) score at admission to the ICU (Knaus et al., 1985), dialysis status, neuromuscular blocker, inhaled nitric oxide, prone positioning, duration between respiratory failure and vv-ECMO setup, blood flow and sweep gas flow on the first day of vv-ECMO support, and ventilator settings at the time of vv-ECMO setup and on days 1, 2, and 3 after vv-ECMO initiation (e.g., PIP; mean airway pressure, MAP; TV; positive end-expiratory pressure, PEEP; respiratory rate, RR; and fraction of inspired O2, FiO_2_). Intubation and extubation, ECMO cannulation and decannulation, ICU and hospital discharge, and time of death were also recorded. Although the effect of ventilator-associated lung injury might be continuous and cumulative during the whole course of mechanical ventilation, inflamed lungs are more subject to mechanical injury during the initial hyperinflammation stage. Similar to previous studies (Schmidt et al., 2019; Chiu et al., 2021), we focused on the impact of ventilator settings during the first three days. Dynamic driving pressure is defined as the difference between PIP and PEEP. Mechanical power during pressure-controlled ventilation is calculated according to the simplified equation: Mechanical power=0.098 ⋅RR ⋅TV ⋅(ΔPinsp+ PEEP), where ΔPinsp is the change in airway pressure during inspiration (Becher et al., 2019).

### Statistical analysis

Data were presented as number of patients (with associated percentage of total patients) or median values (with associated interquartile range), as appropriate. The primary outcome was six-month mortality incidence after the onset of respiratory failure. Mann–Whitney U tests, chi-square tests and Fisher’s exact tests were used for between group comparisons. Covariates presumed to be associated with ARDS mortality based on previous studies, including APACHE II score and Charlson comorbidity index score, as well as covariates with a significant association with mortality in the univariable analysis were subsequently included in the final multivariable Cox regression model (Backward Wald). The following variables were included in the final multivariate Cox regression model: age; sex; body mass index; Charlson comorbidity index score; SOFA score and APACHE II score at admission; use of inhaled nitric oxide, inotropic agents or steroids; prone positioning; neuromuscular blocker use; hemodialysis; arterial blood gas before vv-ECMO setup; and mechanical ventilator settings before vv-ECMO support and during the first three days after vv-ECMO initiation. Among these variables, sex, use of inhaled nitric oxide, inotropic agents or steroids, prone positioning, neuromuscular blocker use, and hemodialysis were categorical variables. The others were continuous variables. We reported hazard ratios and the corresponding 95% confidence intervals. We used the Kaplan–Meier estimator to generate survival curves. *P* values less than 0.05 were considered statistically significant. Statistical analyses were performed using the IBM SPSS software, version 25.

## Results

**Figure 1:**
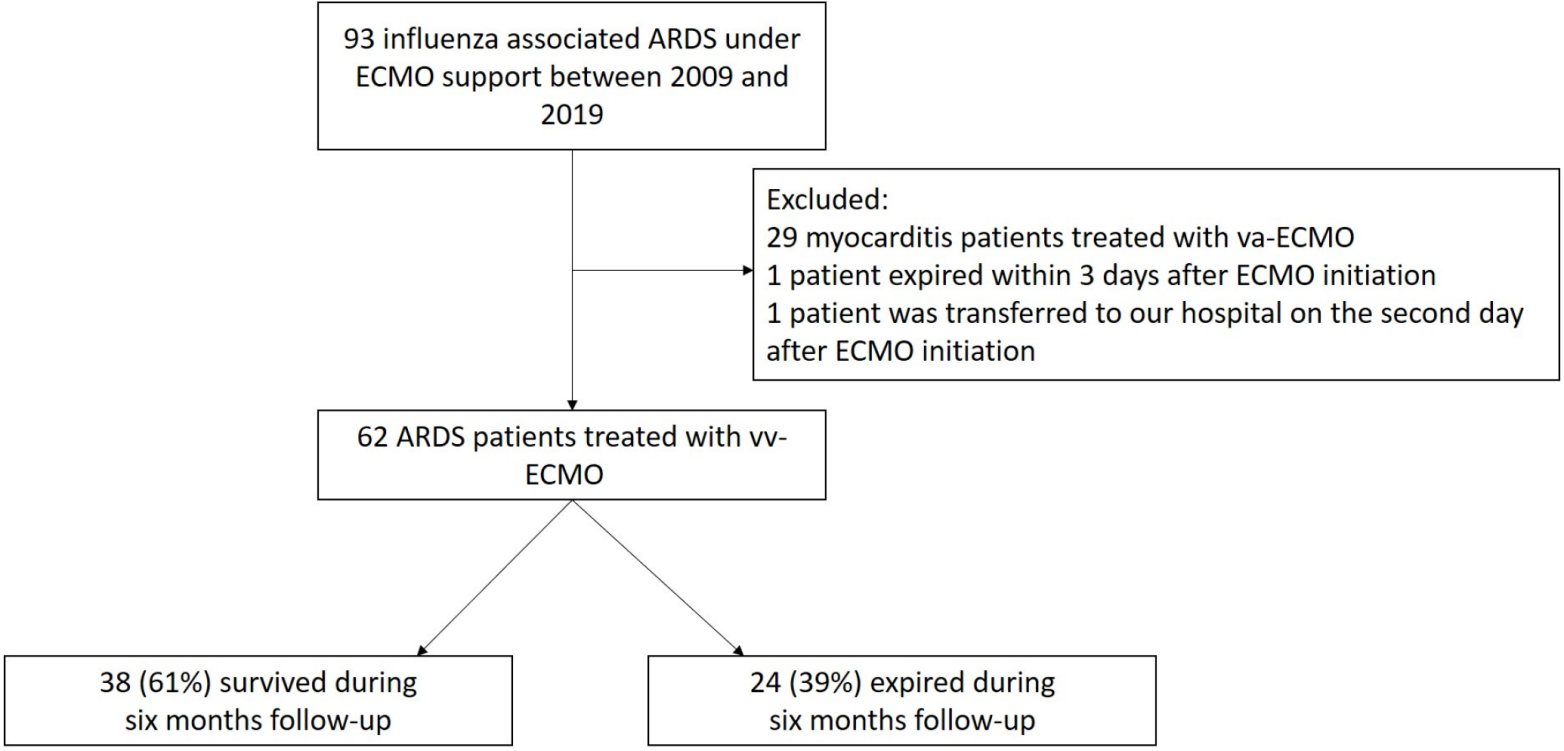
Flow chart depicting the study design. ARDS, acute respiratory distress syndrome; ECMO, extracorporeal membrane oxygenation.

From July 2009 to December 2019, a total of 93 patients with influenza-related ARDS receiving ECMO support were treated in the ICUs of the three study hospitals in Taiwan. Among these, 29 (31%) patients were excluded because they received venoarterial ECMO support for clinically suspected influenza-related myocarditis, 1 (1%) patient was excluded because he died on the day of vv-ECMO initiation, and 1 (1%) patient was excluded because he was transferred to the hospital 2 days after vv-ECMO initiation. After these exclusions, the remaining 62 (67%) patients receiving vv-ECMO support were enrolled in the study (Fig. 1). The clinical and demographic characteristics of the 62 study participants are shown in Table 1. The median patient age was 56 (46–63) years old, 42 participants (68%) were men, 15 (24%) were smokers, and the median Charlson comorbidity index score (Charlson et al., 1987) of the participants was 3 (1–4). Upon admission to the ICU, the median SOFA score was 11 (8–13), and the median APACHE II score was 19 (14–23). The median mean arterial pressure was 85 (70–98) mmHg, and 30 (48%) patients had septic shock.

**Table 1:** Baseline characteristics of patients.

	All (*n* = 62)	Survivors (*n* = 38)	Non-survivors (*n* = 24)	*p*-value
Age, year	56 [46–63]	52 [38–60]	59 [53–65]	0.011
Male	42 (68)	25 (66)	17 (70)	0.679
BMI, kg/m^2^	27.2 [24.1–32.0]	27.4 [24.8–32.6]	26.2 [22.6–31.1]	0.263
SOFA score	11 [8–13]	11 [8 –14]	10 [7–13]	0.119
APACHE II score	19 [14–23]	16 [12–21]	22 [18–28]	<0.001
Charlson comorbidity index score	3 [1–4]	2 [1–3]	4 [2–6]	0.003
Smoking	15 (24)	9 (24)	6 (25)	0.906
Comorbidity				
Diabetes mellitus	16 (26)	10 (26)	6 (25)	0.908
Hypertension	30 (48)	18 (47)	12 (50)	0.840
Cirrhosis	4 (7)	1 (3)	3 (13)	0.289
Coronary artery disease	7 (11)	5 (13)	2 (8)	0.559
Heart failure	4 (7)	1 (3)	3 (13)	0.123
Chronic obstructive pulmonary disease	1 (2)	0	1 (4)	0.387
Chronic kidney disease	3 (5)	2 (5)	1 (4)	>0.999
Mean arterial pressure, mmHg	85 [70–98]	85 [71–98]	84 [70–95]	0.947
Septic shock	30 (48)	17 (45)	13 (54)	0.462
Lactic acid, mmol/L	2.9 [2.1–4.8]	2.8 [2.0–4.3]	3.1 [2.1–5.6]	0.380
Adjunctive therapy				
Prone positioning	5 (8)	2 (5)	3 (13)	0.308
Inhaled nitric oxide	12 (19)	4 (11)	8 (33)	0.027
Neuromuscular blocker	33 (53)	21 (55)	12 (50)	0.686
Arterial blood gas analysis				
PH	7.36 [7.25–7.43]	7.41 [7.31–7.45]	7.29 [7.20–7.38]	0.002
PaO_2_, mmHg	59 [44–65]	58 [44–68]	59 [43–64]	0.680
PaCO_2_, mmHg	38 [33–54]	35 [31–40]	50 [41–63]	<0.001
HCO_3_^−^, mmol/L	23 [19–26]	22 [19–24]	24 [20–28]	0.092
Mechanical ventilation parameters before ECMO support				
FiO2, %	1.0 [1.0–1.0]	1.0 [1.0–1.0]	1.0 [1.0–1.0]	0.614
Tidal volume, mL/kg	6.9 [5.5–9.2]	6.7 [5.5–9.2]	7.2 [6.2–9.2]	0.578
PEEP, cmH_2_O	12 [10–15]	12 [10–15]	14 [10–14]	0.777
PIP, cmH_2_O	29 [26–32]	28 [26–32]	31 [28–33]	0.653
MAP, cmH_2_O	19 [16–22]	19 [17–22]	20 [16–23]	0.943
Respiratory rate, /min	21 [16–28]	20 [16–27]	21 [18–30]	0.702
Dynamic driving pressure, cmH_2_O	17 [14–21]	16 [12–21]	17 [14–21]	0.386
PaO_2_/FiO_2_	60 [45–73]	59 [44–74]	62 [50–73]	0.734
RESP score	3 [0–4]	4 [1–5]	1 [−2–3]	0.003
Duration from respiratory failure to ECMO support, hours	22 [6–54]	17 [6–41]	30 [6–132]	0.080

**Notes.**

Data are expressed as number (%) or median (25th–75th percentiles).

SOFA Sequential Organ Failure Assessment APACHE Acute Physiology and Chronic Health Evaluation PEEP positive end expiratory pressure PIP peak inspiratory pressure MAP mean airway pressure RESP Respiratory Extracorporeal Membrane Oxygenation Survival Prediction ECMO extracorporeal membrane oxygenation

The median duration from receiving mechanical ventilation to ECMO initiation was 22 (6–54) hours. Before ECMO, adjunctive therapies were used in 15 (24%) patients, prone positioning was used with 5 (8%) patients and 12 (19%) patients received inhaled nitric oxide. A neuromuscular blocker was administered to 33 (53%) patients.

The pressure control mechanical ventilation mode was used in these ARDS patients before and during ECMO. Before ECMO initiation, the median tidal volume was 6.9 (5.5–9.2) mL/kg predicted body weight, the median respiratory rate was 21 (16–28) breaths/min, the median PEEP level was 12 (10–15) cmH_2_O, and the median PIP was 29 (26–32) cmH_2_O.

After ECMO initiation (Table 2), 94% of patients were paralyzed using a neuromuscular blocker. The remaining 6% patients were sedated using benzodiazepine or propofol. Median blood flow was 3.0 (2.5–3.4) L/min and median sweep gas flow was 3.0 (2.0–3.0) L/min. From day 1 to day 3, the mean tidal volume was reduced to 5.1 (4.0–6.4) mL/kg predicted body weight and the mean respiratory rate was reduced to 13 (12–17) breaths/min, with a mean PEEP of 12 (10–14) cmH_2_O. Mean dynamic driving pressure was reduced from 17 (14–21) to 15 (13–17) cmH_2_O, and mean mechanical power was decreased from 23.9 (16.9–32.9) to 11.5 (8.3–15.8) J/min. Mean PaCO_2_ decreased from 38 (33–54) to 31 (27–37) mmHg, and mean PaO_2_ improved from 59 (44–65) to 73 (61–87) mmHg.

**Table 2:** Characteristics and management during extracorporeal membrane oxygenation support.

	All (*n* = 62)	Survivors (*n* = 38)	Non-survivors (*n* = 24)	*p*-value
ECMO setting				
Blood flow, L/min	3.0 [2.5 –3.4]	3.0 [2.5–3.3]	3.0 [2.6–3.7]	0.255
Sweep gas flow, L/min	3.0 [2.0 –3.0]	3.0 [2.0–4.0]	3.0 [2.0 –3.0]	0.451
Arterial blood gas analysis				
pH	7.44 [7.41 –7.50]	7.44 [7.41–7.49]	7.45 [7.40–7.50]	0.679
PaO_2_, mmHg	73 [61–87]	72 [60–80]	75 [66–93]	0.189
PaCO_2_, mmHg	31 [27–37]	31 [26–37]	31 [28–38]	0.958
HCO_3_^−^, mmol/L	21 [20–25]	21 [20–25]	21 [19–25]	0.802
Adjunctive therapy				
Steroid	21 (34)	9 (24)	12 (50)	0.033
Dialysis	25 (40)	12 (32)	13 (54)	0.077
Neuromuscular blocker	58 (94)	34 (90)	24 (100)	0.100
Mechanical ventilation parameters from day 1 to day 3 after ECMO support				
FiO2	0.6 [0.5–0.7]	0.6 [0.4–0.7]	0.6 [0.4–0.7]	0.198
Tidal volume, mL/kg	5.1 [4.0–6.4]	5.1 [3.9–6.4]	5.2 [4.0–6.2]	0.833
PEEP, cmH_2_O	12 [10–14]	11 [10–14]	12 [11–14]	0.538
PIP, cmH_2_O	27 [25–29]	28 [25–29]	26 [25–28]	0.257
MAP, cmH_2_O	17 [14–18]	15 [14–18]	17 [15–19]	0.061
Respiratory rate, /min	13 [12–17]	12 [12–15]	15 [13–18]	0.013
Dynamic driving pressure, cmH_2_O	15 [13–17]	16 [13–17]	14 [13–16]	0.146
Mechanical power, J/min	11.5 [8.3–15.8]	11.3 [7.7–14.7]	11.8 [8.3–18.1]	0.352
Decrease of mechanical power, J/min	12.5 [4.4–21.4]	12.7 [5.2–23.8]	12.2 [3.4–19.9]	0.562
Decrease of lactic acid, mmol/L				
First day	0.7 [0.2–1.9]	0.7 [0–2.1]	0.6 [−0.2–1.8]	0.765
Second day	1.0 [0.2–2.4]	1.2 [0.2–3.0]	0.7 [−0.7–2.1]	0.159
Third day	1.0 [0.3–2.4]	1.4 [0.5–2.8]	0.9 [0.2–1.8]	0.208
Complications	13(21)	6 (16)	7 (29)	0.208
Gastrointestinal bleeding	6 (10)	2 (5)	4 (17)	
Intracranial hemorrhage	1 (2)	1 (3)	0 (0)	
Surgical site bleeding	5 (8)	3 (8)	2 (8)	
Other bleeding	2 (3)	1 (3)	1 (4)	
Concomitant infection	37 (60)	21 (55)	16 (67)	0.373
*Aspergillus* spp*.*	1 (2)	0 (0)	1 (4)	
*Klebsiella pneumoniae*	7 (11)	5 (13)	2 (8)	
*Pseudomonas aeruginosa*	2 (3)	1 (3)	1 (4)	
*Acinetobacter baumannii*	18 (29)	10 (26)	8 (33)	
*Staphylococcus aureus*	4 (7)	2 (5)	2 (8)	
*Escherichia coli*	4 (7)	2 (5)	2 (8)	
*Streptococcus* spp.	3 (5)	1 (3)	2 (8)	
Others	24 (39)	13 (34)	11 (46)	
Duration of ECMO (days)	14 [7–20]	10 [7–18]	17 [8–25]	0.244
Duration of mechanical ventilator (days)	23 [15–38]	22 [15–38]	23 [14–38]	0.908
Length of ICU stay (days)	23 [16–41]	24 [16–44]	23 [13–38]	0.492
Length of hospital stay (days)	36 [22–64]	40 [27–78]	24 [14–60]	0.036

**Notes.**

Data are expressed as number (%) or median (25th–75th percentiles).

PEEP positive end expiratory pressure PIP peak inspiratory pressure MAP mean airway pressure ECMO extracorporeal membrane oxygenation

A total of 21 (34%) patients received steroids, and 25 (40%) patients underwent hemodialysis. Overall, 13 (21%) patients had one or more ECMO-associated complications, and 37 (60%) patients had secondary bacterial infections, with the most common pathogen being *Acinetobacter baumannii*. The median duration of ECMO for all 62 patients was 14 (7–20) days, median mechanical ventilator duration was 23 (15–38) days, the median ICU stay was 23 (16–41) days, and the median hospital stay was 36 (22–64) days.

Twenty-four patients (39%) died within the first six months after respiratory failure. All 24 of these patients died in the ICU. The most common cause of death was secondary bacterial pneumonia complicated by septic shock (75%). Compared with non-survivors, survivors were younger (52 *vs*. 59, *p* = 0.011) and had lower initial APACHE II scores (16 *vs*. 22, *p* < 0.001) and Charlson comorbidity index scores (2 *vs*. 4, *p* = 0.003). Inhaled nitric oxide and steroid use was lower in the survivor group compared to the non-survivor group.

**Figure 2:**
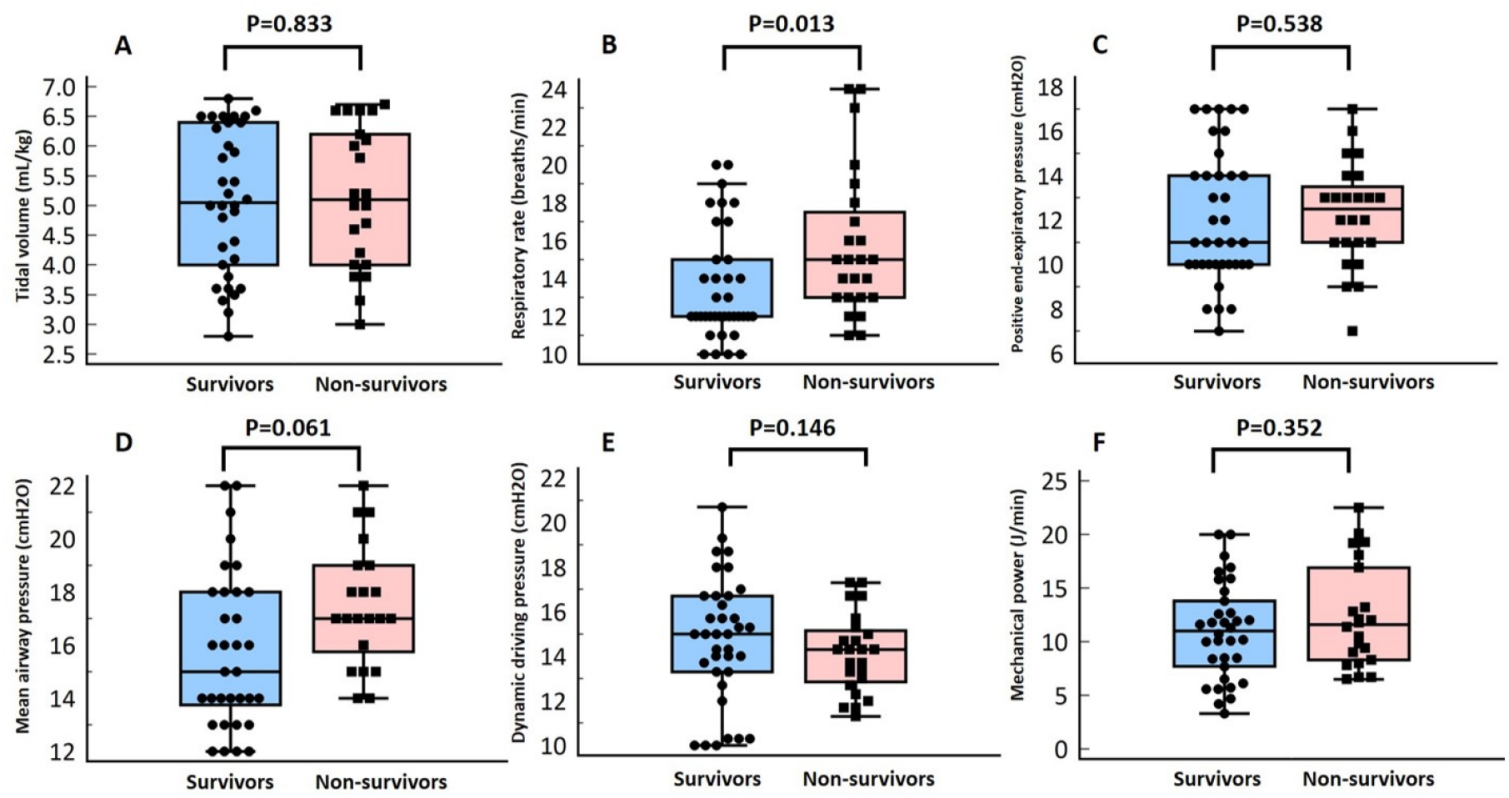
Box-plot of mean tidal volume per predicted body weight (A), mean respiratory rate (B), mean mean airway pressure (C), mean positive end-expiratory pressure (D), mean dynamic driving pressure (E), and mean mechanical power (F) from day 1 to day 3 after venovenous extracorporeal membrane oxygenation (vv-ECMO) support according to the six-month outcome.

Compared with non-survivors, during vv-ECMO support, survivors had a similar blood flow (3.0 vs. 3.0 L/min, *p* = 0.255), sweep gas flow (3.0 vs. 3.0 L/min, *p* = 0.451), mean tidal volume from day 1 to day 3 (5.1 *vs.* 5.2 mL/kg predicted body weight, *p* = 0.833), mean dynamic driving pressure (15 *vs.* 14 cmH2O, *p* = 0.146), mean mechanical power (11.3 *vs.* 11.8 J/min, *p* = 0.352), and mean PEEP (11 *vs.* 12 cmH2O, *p* = 0.538), but survivors had a lower mean respiratory rate (12 *vs*. 15, *p* = 0.013; Table 2 and Fig. 2). Using the receiver operating characteristic curve analysis (area under curve=0.69, 95% CI = 0.56−0.82), a cutoff point with a respiratory rate of 12 breaths/min could predict six-month mortality with 83% sensitivity and 57% specificity.

**Figure 3:**
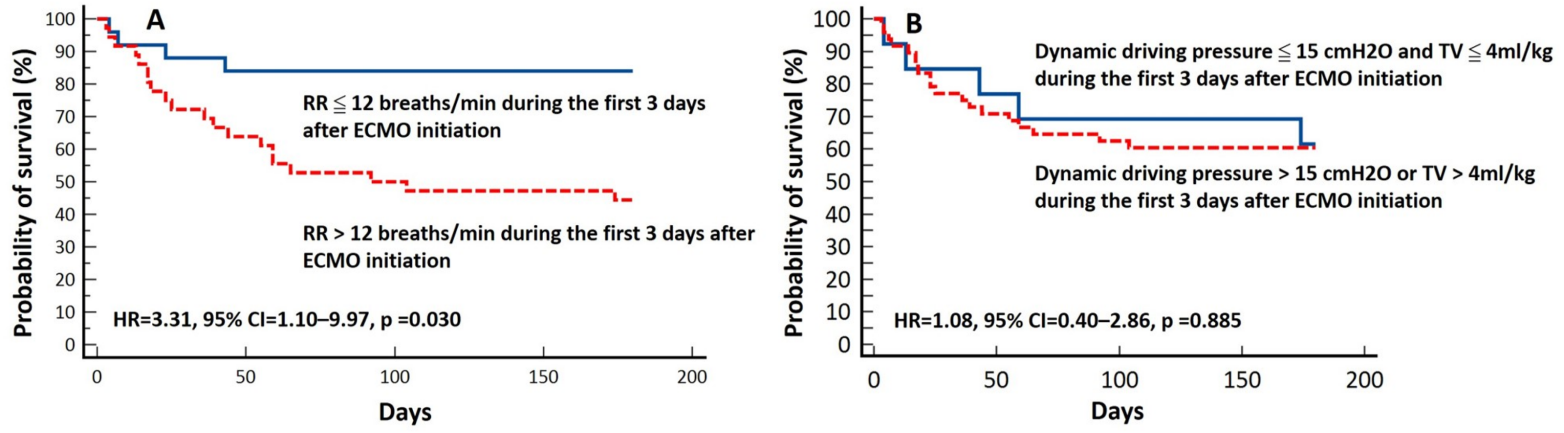
Kaplan–Meier plot and log-rank test for six-month survival according to respiratory rate (RR) (A), tidal volume (TV) and dynamic driving pressure (B) during the first 3 days after venovenous extracorporeal membrane oxygenation (vv-ECMO) initiation.

**Figure 4:**
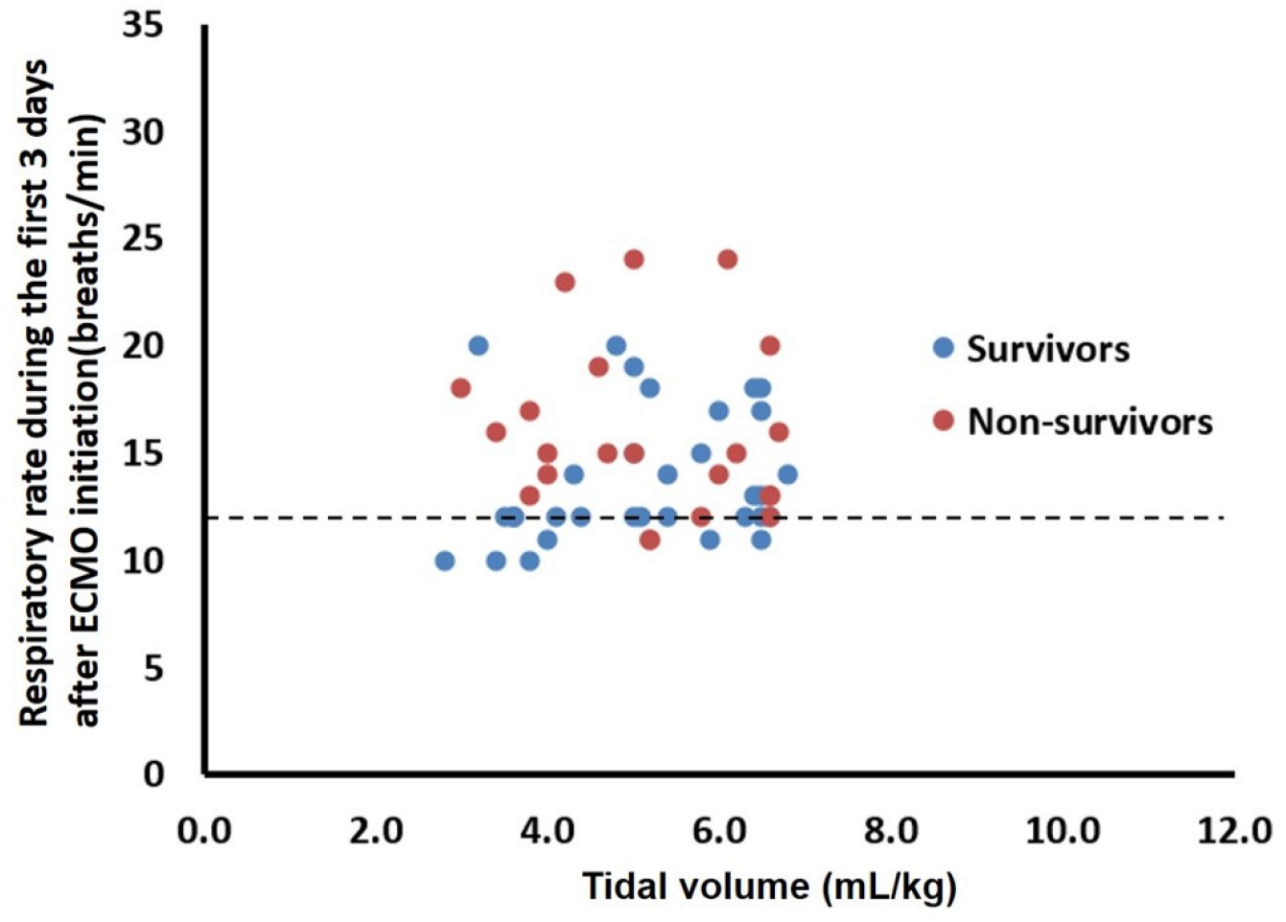
Distribution of respiratory rate versus tidal volume during the first 3 days after venovenous extracorporeal membrane oxygenation (vv-ECMO) support for each patient. Twenty five patients (mortality 16%) fell within the limits, which defined as respiratory rate ≦12 breaths/min.

A univariate Cox proportional hazards regression analysis (Table 3) showed that older age (hazard ratio, HR = 1.05, 95% confidence interval, 95% CI = 1.01–1.1; *p* = 0.018), more severe respiratory acidosis before vv-ECMO support (HR = 1.04, 95% CI=1.02–1.06; *p* < 0.001), use of inhaled nitric oxide (HR = 2.49, 95% CI = 1.07–5.84; *p* = 0.035), and use of steroids (HR = 2.52, 95% CI = 1.13–5.62; *p* = 0.024) were associated with increased mortality. The Kaplan–Meier analysis showed that patients with a mean respiratory rate >12 breaths/min on the first 3 days after ECMO support had a higher six-month mortality rate (log-rank test, *p* = 0.003; Fig. 3A). Furthermore, the multivariable Cox proportional hazards regression analysis showed that a respiratory rate higher than 12 breaths/min (HR = 3.31, 95% CI = 1.10–9.97, *p* = 0.034), as an independent factor, was associated with six-month mortality (Table 3 and Fig. 4).

**Table 3:** Univariable and multivariable Cox proportional hazards regression analysis to predict six-month mortality in patients with severe acute respiratory distress syndrome with extracorporeal membrane oxygenation support.

	Univariable			Multivariable		
	Hazard ratio	95% CI	*p*-value	Hazard ratio	95% CI	*p*-value
Age, per additional year	1.05	1.01–1.10	0.018	1.06	1.01–1.12	0.015
PaCO_2_, mmHg	1.04	1.02–1.06	<0.001	1.04	1.02–1.07	<0.001
Inhaled nitric oxide	2.49	1.07–5.84	0.035			
Steroid exposure	2.52	1.13–5.62	0.024			
Mean respiratory rate>12/min on the first 3 days after ECMO setup	4.44	1.51–13.02	0.007	3.31	1.10–9.97	0.034

**Notes.**

CI confidence interval

## Discussion

In this multicenter retrospectively study, we found that a lower respiratory rate setting during vv-ECMO support was significantly associated with better survival among patients with influenza-associated severe ARDS. In addition to tidal volume and driving pressure, we suggest monitoring respiratory rate during ECMO support. The respiratory rate should be kept low.

The goal of mechanical ventilation in patients with ARDS is to maintain adequate gas exchange, but there are also many well-known complications of mechanical ventilation such as barotrauma, atelectrauma, oxygen toxicity, and hemodynamic compromise (Fan, Brodie & Slutsky, 2018; Slutsky & Ranieri, 2013). Vv-ECMO support during mechanical ventilation enables patients to ventilate with a very low tidal volume, low plateau pressure, and low respiratory rate, thereby minimizing ventilation-induced lung injury (Schmidt et al., 2019). However, the ideal mechanical ventilator settings for ECMO initiation are still not well defined (Peek et al., 2009; Extracorporeal Life Support Organization, 2017; Combes et al., 2018). Using a rat model of acid-induced lung injury, Frank et al. (2002) showed that a tidal volume reduction from 12 to six to three mL/kg, with the same level of PEEP (10 cmH2O), decreased pulmonary edema and lung injury. Recently, Del Sorbo et al. (2020) demonstrated that there was a linear relationship between the change in driving pressure and the plasma concentration of inflammatory mediators. The systemic inflammatory biomarkers increased with larger increases in inspiratory pressure in patients with severe ARDS on vv-ECMO, therefore, an ultra-protective lung ventilation strategy during ECMO support was proposed (Schmidt et al., 2019) that set the tidal volume less than four mL/kg or driving pressure less than 15 cmH_2_O, plateau pressure less than 25 cmH_2_O, and PEEP greater than 10 cmH_2_O (Schmidt et al., 2019; Rozencwajg et al., 2019). Rozencwajg et al. found that ultra-protective ventilation for patients with vv-ECMO was associated with significantly decreased plasma sRAGE and other cytokine concentrations, which were biomarkers of lung injuries (Rozencwajg et al., 2019). Pham et al. (2013) studied 123 patients with influenza A (H1N1)-induced ARDS treated with ECMO and found that a reduction in plateau pressure (25 *vs*. 29, *p* < 0.01) on the first day of ECMO was significantly associated with survival.

Although the Extracorporeal Life Support Organization guidelines for patients with respiratory failure receiving ECMO support recommend a lung rest strategy (FiO2 <0.4, plateau pressure <25 cmH2O) (Extracorporeal Life Support Organization, 2017), a recent study by Schmidt et al. (2019) demonstrated a lack of association between mechanical ventilator settings during the first two days of ECMO and survival. In our study, we also did not find a significantly lower mortality rate among the 13 patients receiving ultra-protective ventilation (Fig. 3B) compared to those without ultra-protective ventilation (39% *vs*. 40%, *p* = 0.941). An animal study (Araos et al., 2019) found that a near-apneic ventilatory strategy, which sets the respiratory rate at five breaths/min, significantly decreased histologic lung injury and fibroproliferation compared with conventional protective lung strategies. Previous experimental studies have also shown that decreasing the respiratory rate may prevent ventilator-induced lung injury (Hotchkiss Jr et al., 2000; Retamal et al., 2016).

An analysis from a pooled database of 4,549 patients with ARDS found that higher respiratory rates could be injurious (Costa et al., 2021). A recent review by Abrams et al. also suggested setting the initial respiratory rate of ≤ 10 breaths/min during ECMO for ARDS (Abrams et al., 2020) adding that the recommendation for a respiratory rate below the lower limit of the EOLIA protocol is based on the presumption that lower respiratory rates may prevent lung injury (Abrams et al., 2020; Combes et al., 2018). In our study, we also found that a lower respiratory rate during vv-ECMO support was significantly associated with lower mortality, which supports the hypothesis that a near-apneic strategy with a low respiratory rate during vv-ECMO support could improve outcomes. Accordingly, to achieve lung protection for patients receiving vv-ECMO for ARDS, gas exchange could be primarily supported by the ECMO. In our opinion, optimal ventilator settings during ECMO should be guided by best available evidence from previous studies, such as the ultra-protective strategy. (Schmidt et al., 2019; Extracorporeal Life Support Organization, 2017) The initial settings could be as follows: Tidal volume ≤ 4 ml/kg, driving pressure ≤ 15 cmH2O, inspiratory plateau pressure <25 cmH2O, FiO2 <0.4 and PEEP ≥ 10 cmH2O. Furthermore, based on our study results, respiratory rate should be set lower than 12 breaths per minute.

Similar to patient populations in previous studies, our patients with influenza-associated ARDS treated with vv-ECMO (Roch et al., 2010; Davies et al., 2009; Pham et al., 2013; Patroniti et al., 2011) were relatively young, not obese, and had extensive viral pneumonia with extremely severe lung injury. However, some differences should be highlighted: less patients in this study received inhaled nitric oxide or prone positioning (19% and 8%, respectively) before vv-ECMO support compared with patients in previous studies from Australia (32%), New Zealand (20%) (Davies et al., 2009), and France (72% and 45%) (Pham et al., 2013); and the duration of mechanical ventilation before ECMO was shorter in our study (22 h) than in previous studies (from 2 to 4 days) (Noah et al., 2011; Davies et al., 2009; Pham et al., 2013; Patroniti et al., 2011). This finding suggests that vv-ECMO is more readily available as a rescue therapy in Taiwan than it is in other countries. In a previous cohort study, ECMO outcomes were highly variable with short-term mortality ranging from 29% to 56% (Roch et al., 2010; Noah et al., 2011; Davies et al., 2009; Pham et al., 2013; Patroniti et al., 2011), reflecting heterogeneity in the patient population, disease severity, and treatment received.

Initiation of antiviral treatment is recommended for patients with influenza. Oseltamivir administration within 48 h of symptom onset has been demonstrated to improve outcomes (Hernu et al., 2018). Conversely, steroid use in influenza patients has consistently been found to be predictive of poor outcomes in several studies, and a meta-analysis by Ni et al. (2019) showed that steroid use could increase mortality (risk ratio = 1.75, 95% CI [1.30–2.36], *p* = 0.0002). Consistent with the results from previous studies, we found that steroid use was associated with poor outcomes (HR = 2.29, 95% CI=1.06−4.96, *p* = 0.04).

This study has several limitations. First, there was no universal ventilator protocol for patients on ECMO. The primary attending physicians adjusted ventilator settings according to the patient’s clinical condition after ECMO initiation. Respiratory rate might be a marker of disease severity in ARDS but not a causal factor. Second, our study may be affected by type I error inflation because of the multiple comparisons used in our analysis, so our results should be interpreted with caution. Third, the sample size was small and the possibility of unobserved confounders explaining the differences in outcomes cannot be eliminated.

## Conclusions

Patients with influenza-related severe ARDS receiving vv-ECMO support have a high mortality rate. The findings from this multicenter retrospective study suggest that patients with lower respiratory rates during vv-ECMO support have better outcomes. However, owing to the limitations of the study, it is necessary to conduct a prospective randomized control trial to verify these findings.

##  Supplemental Information

10.7717/peerj.14140/supp-1 Supplemental Information 1 Raw data

10.7717/peerj.14140/supp-2 Supplemental Information 2 CodeBook

## Additional Information and Declarations

### Data Availability

The following information was supplied regarding data availability: The raw data is available in the Supplemental Files.
